# Psoralen: a narrative review of current and future therapeutic uses

**DOI:** 10.1007/s00432-024-05648-y

**Published:** 2024-03-15

**Authors:** Panagis Galiatsatos, Daniella D. Maydan, Elle Macalpine, Beatrice Schleupner, Alexandra Hunter Aitchison, Andrew D. Lerner, Benjamin Levy, Aditya Halthore, William Eward

**Affiliations:** 1 The Sidney Kimmel Comprehensive Cancer Center, the Johns Hopkins School of Medicine, 4940 Eastern Avenue, 4th Floor, Asthma & Allergy Building, Baltimore, MD 21224 USA; 2 Division of Pulmonary and Critical Care Medicine, Department of Medicine, Johns Hopkins School of Medicine, Baltimore, MD USA; 3 Department of Orthopaedic Surgery, Washington University in St. Louis, St. Louis, MO USA; 4 Department of Orthopaedic Surgery and Duke Cancer Institute, Duke University, Durham, NC USA; 5 Department of Radiation Oncology and Molecular Radiation Sciences, Johns Hopkins School of Medicine, Baltimore, MD USA

**Keywords:** Psoralen, Photoactivation, Cancer Treatment, Immunotherapy

## Abstract

Psoralen is a family of naturally occurring photoactive compounds found in plants that acquire potential cytotoxicity when activated by specific frequencies of electromagnetic waves. Psoralens penetrate the phospholipid cellular membranes and insert themselves between the pyrimidines of deoxyribonucleic acid (DNA). Psoralens are initially biologically inert and acquire photoreactivity when exposed to certain classes of electromagnetic radiation, such as ultraviolet light. Once activated, psoralens form mono- and di-adducts with DNA, leading to marked cell apoptosis. This apoptotic effect is more pronounced in tumor cells due to their high rate of cell division. Moreover, photoactivated psoralen can inhibit tyrosine kinase signaling and influence the immunogenic properties of cells. Thus, the cytotoxicity of photoactivated psoralen holds promising clinical applications from its immunogenic properties to potential anti-cancer treatments. This narrative review aims to provide an overview of the current understanding and research on psoralen and to explore its potential future pharmacotherapeutic benefits in specific diseases.

## Introduction

Psoralen is a naturally occurring phytoalexin found in the seeds of Psoralea corylifolia plants and certain fruits, such as figs and citrus fruits, whose chemical structure resembles that of coumarin due to the addition of a fused furan ring (Ruan et al. 2007). Psoralen’s clinical use dates back to 2000 BC in ancient Egypt and India, where it was utilized in the treatment of various skin conditions due to its photosensitive properties (Lim et al. 2005). In those early applications, preparations containing psoralen were either ingested or applied topically to the skin. Subsequently, individuals would expose themselves to sunlight to activate the therapeutic compounds (Lim et al. 2005; Perrine et al. 2007). Psoralen has been available in the United States since the mid-twentieth century, with first reports indicating the use of ultraviolet A (UVA) light for photoactivation (Lim et al. 2005; Perrine et al. 2007; Bethea et al. 1999). This review will explore the mechanism of activity, established clinical utility, current investigation, and potential future uses of photoactivated psoralens.

## Mechanism of activity

**Fig. 1:**
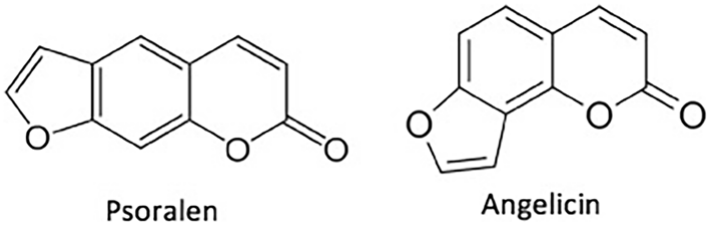
Chemical structure of Psoralen and Angelicin, with figure from Melough et al. (2018)

Phototherapy using furocoumarins, such as psoralen (linear furocoumarin) and angelicins and allosporalens (angular fuorocoumarins) (Fig. 1), have been extensively known and utilized (Pathak and Fitzpatrick 1992; Melough et al. 2018). The phototherapeutic activity of these compounds arises mainly from their ability to form adducts with the pyrimidine bases of DNA (Seret et al. 1992). The formation of these adducts and oxygen activated species may also be considered responsible for most of the negative effects of the furocoumarin therapies to date. Such a basis will be reviewed in an effort to understand the therapeutic impact of furocoumarins through their mechanism of activity, specifically psoralen, while also introducing how such a biologic action may result in undesirable health outcomes.

**Fig. 2:**
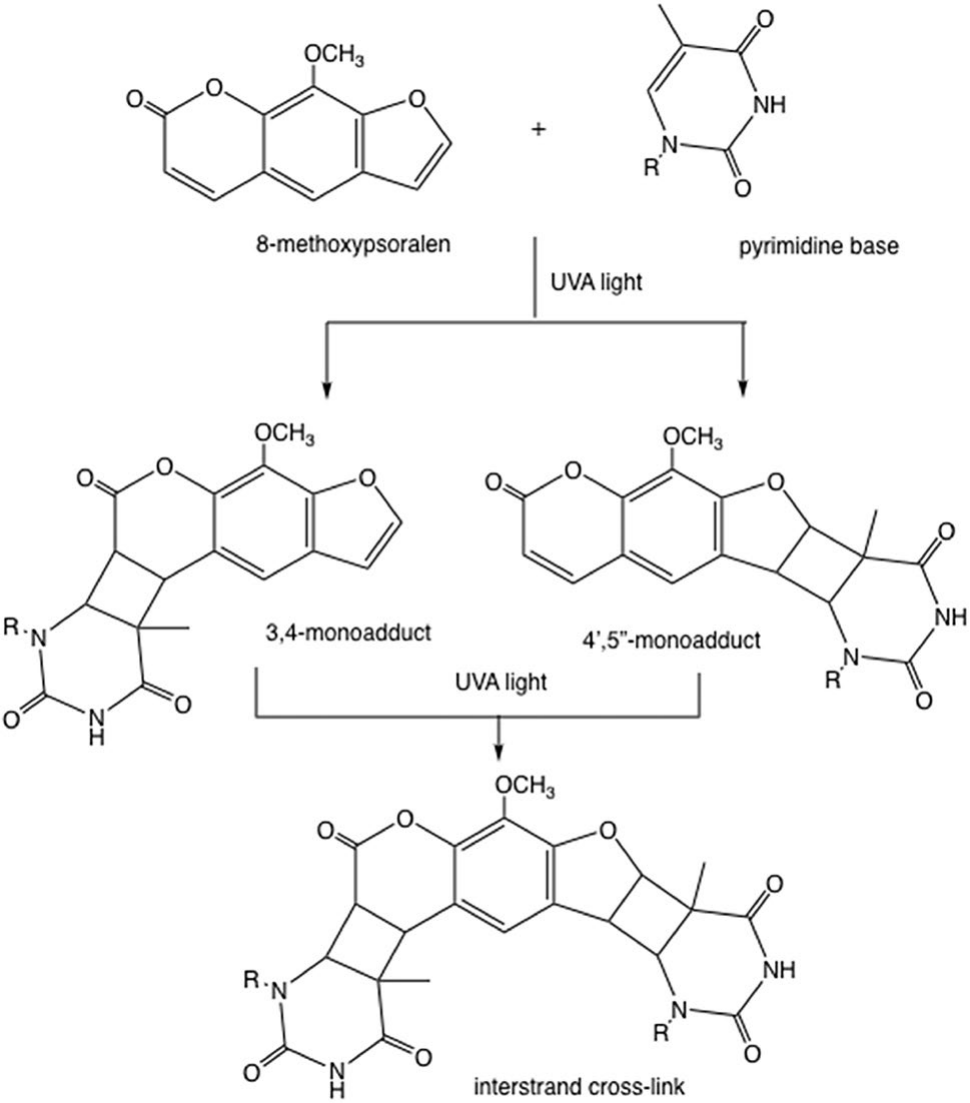
DNA cross-linking via activated 8-methoxypsoralen. This compound can interact with a thymine base to create either a 3,4-mono-adduct or a 4′,5′-monoadduct. Upon exposure to a second dose of ultraviolet-A (UVA) light, these mono-adducts have the potential to create a second crosslink with another thymine base on the opposing DNA strand (Hamblin and Abrahamse 2020)

Psoralens are utilized in various treatment modalities, commonly in conjunction with ultraviolet light exposure (UVA), in a therapeutic process known as PUVA therapy. They can be applied topically via creams or lotions for localized skin conditions such as psoriasis and vitiligo, where direct application ensures targeted treatment of affected areas. Alternatively, oral ingestion of psoralen pills offers a systemic treatment approach, suitable for extensive or refractory skin disorders. Although less frequent, parenteral injections of psoralens are available for specific cases requiring a different administration route. After administration, psoralens can pass through the phospholipid bilayer of cells and insert themselves in between DNA’s pyrimidine bases. Without activation, they remain biologically inert and are excreted within 24 h without any significant physiologic sequelae (Bethea et al. 1999). However, if psoralens are activated by absorbing the energy from photons of UVA light, whose frequency corresponds to the energy required to initiate the photochemical reaction, they form mono- and di-adducts with the DNA (Fig. 2; Hamblin and Abrahamse 202; Gasparro et al. 1997). Photoactivated psoralen is thought to then induce DNA modification, along with subsequent modification of lipids and proteins within the cell and at the cell surface (Gasparro et al. 1997; Berger et al. 2002). These changes result in enhanced targeted cellular immunogenicity, leading to the eventual elimination of the affected cell (Bethea et al. 1999; Gasparro et al. 1997; Berger et al. 2002; Schmitt et al. 1995). Furthermore, changes to antigen presentation and cytokine secretion patterns have been recognized to contribute to psoralen’s therapeutic effects regarding targeted cell elimination (Gasparro et al. 1997).

The cascade of events following photoreactive psoralen-induced DNA damage includes activation of p53 and upregulation of p21waf/Cip, which lead to apoptosis (Schmitt et al. 1995; El-Domyati et al. 2013). Cellular death may also result from activated psoralen’s effects on tyrosine kinase signaling, impacting various components necessary for cellular stability, growth, and signaling (Xia et al. 2014). Cells with aberrant or high rates of cellular division are more selectively targeted by photoactivated psoralen, resulting in the initiation of apoptosis in cells at the core of many diverse pathologies, such as cancers and autoimmune conditions. The long-lasting immunogenic effects of psoralen are attributed to the acute photoadduct DNA damage it causes (Lim et al. 2005; Bethea et al. 1999). This DNA damage, triggered by photoactivated psoralen, leads to the generation of apoptotic cells. These cells are then phagocytosed by immature dendritic cells, a process that can be influenced by, but not soley dependent on the presence of inflammatory cytokines (Xia et al. 2014; Wang et al. 2016; Albert et al. 1998). This uptake by dendritic cells initiates a cascade of immune responses, as these cells perform their sentinel-like role in maintaining an individual's homeostatic health (O'Keeffe et al. 2015). Through this cell-mediated immunity, specific antigens associated with diseased cells, including those present in tumors or autoimmune diseases, can be recognized (O'Keeffe et al. 2015). Consequently, the immediate actions of psoralen are complemented by a lasting immune effect, potentially providing ongoing protection beyond initial treatments.

While such biologic activity of psoralen is promising towards targeting diseases and their aberrant molecular mechanisms, it must be noted that the same mechanism of activity may lead to negative sequelae. Specifically, given psoralen’s influence on DNA integrity through activation of p53 and upregulation of p21waf/Cip, it is plausible that psoralen in of itself poses a risk for cancer development, specifically cutaneous malignanicies (Geller et al. 2018; Stern and Study 2001; Lunder and Stern 1998; Kang et al. 2014). A challenge with reaffirming biological influence of psoralen to these outcomes is confounded by two factors. First, psoralen is often used in treatments for diseases that are known to be associated with malignancies (Vaengebjerg et al. 2020). Next, psoralen is often used in conjunction with other therapies for disease treatment, therapies in of themselves linked to cancer (e.g., methotrexate, TNF-alpha inhibitors) (Geller et al. 2018; Inose et al. 2020). Regardless of such potential confounding concerns, there continues to be statistical findings showing psoralen’s clinical significance in its association with cancer genesis, along with other potential toxicities (e.g., hepatotoxicity) whose mechanisms are still unknown (Ren et al. 2020).

## Clinical review

The acute and long-term effects of psoralens have also been observed in clinical settings. Psoralens, are utilized in various treatment modalities, commonly in conjunction with UVA exposure, in a therapeutic process known as PUVA (Psoralen + UVA) therapy. They can be applied topically via creams, lotions, or baths for localized skin conditions such as psoriasis and vitiligo, where direct application ensures targeted treatment of affected areas. Alternatively, oral ingestion of psoralen pills offers a systemic treatment approach, suitable for extensive or refractory disorders. Lastly, parenteral injections of psoralens are available for specific cases requiring a different administration route. Regardless of the method, the activation of psoralens through subsequent UVA exposure is critical to achieving the desired therapeutic effect in treating various conditions. Parrish et al. reported on the first use of PUVA therapy to treat psoriasis in 1974 (Parrish et al. 1974). Since then, psoralens, such as methoxsalen, have been extensively studied for their pharmacotherapeutic potential in treating specific oncological, hematological, and autoimmune diseases (Perrine et al. 2007). Methoxsalen has been used clinically for the treatment of cutaneous T cell lymphoma (CTCL), where long-term responses were seen in the subset of patients who had both an acute clearing of their lymphoma and continued remission decades later (Edelson et al. 1987). Here, we explore various ongoing and potential future uses of photoactive psoralens.

### Cancer

Psoralen has demonstrated its utility in the treatment of various cancers, leveraging its photoreactive properties and specific targeting of highly active, replicating cells. The initial case report detailing the use of Psoralen in cancer therapy was published in 1976. This report described the administration of oral methoxsalen to individuals diagnosed with mycosis fungoides, a variant of CTCL (Gilchrest et al. 1976). After ingesting psoralen medication, patients were exposed to long-wave ultraviolet light. Remarkably, some patients experienced complete clearing of the lymphoma as early as 4 weeks (after 12 treatments) (Gilchrest et al. 1976). Over the next few decades, PUVA has consistently demonstrated its effectiveness in treating mycosis fungoides, with response rates ranging from 65 to 85% in patients diagnosed with early stages of this malignancy (Olsen et al. 2016). More recent studies have indicated that low-dose oral PUVA and bath-PUVA therapy continue to be effective for mycosis fungoides, particularly in the early stages (Vieyra-Garcia et al. 2019; Shintani et al. 2022). Moreover, long-term follow-up for other CTCLs have revealed promising outcomes in both initial treatment response and remission for patients treated with PUVA (Knobler et al. 2012). Phototherapy-based treatments with ionizing radiation do increase the risk of generating reactive oxygen species and skin malignancies (Oulee et al. 2023). However, providers can adjust phototherapy on a case-by-case basis using the patient’s skin pigmentation and their genetic likelihood for photoadaption to reduce the risk of accumulating mutations (Oulee et al. 2023). The tolerability and safety profiles of psoralen in these patients have been well documented, suggesting that the therapeutic ratio of a combination of psoralen with electromagnetic radiation may be higher than the therapeutic ratio for more conventional, established oncologic therapies.

Psoralen treatments have also shown therapeutic advantages in the context of melanoma. In an in vitro study using a human melanoma cell line, different types of psoralens combined with ultraviolet light exhibited significant cytotoxicity against the cancer cells (Carneiro Leite et al. 2004; Oldham et al. 2016). Although these findings were observed in vitro using human melanoma cells, psoralen’s treatment potential is on par with that seen in prior cutaneous malignancies (Carneiro Leite et al. 2004). Several animal studies have demonstrated the therapeutic benefits of using psoralen to treat malignancy. In mouse models, mice with syngeneic 4T1 tumors treated with psoralen and electromagnetic radiation showed a significant reduction in tumor burden compared to mice injected with a placebo (Oldham et al. 2016). Furthermore, in vitro studies have shown the viability of psoralen photoactivation in obtaining a measurable antitumor response (Oldham et al. 2016). However, the treatment of deep-seated solid tumors poses a potential difficulty given ultraviolet radiation’s inability to penetrate into deeper tissues. In previous clinical trials, PUVA therapy has been demonstrated to have a maximum penetration distance of < 1 mm. Therefore, ultraviolet radiation is unlikely to activate psoralen for solid tumors deeper than 1 mm.

In response to this limitation, studies have begun to look at different classes of electromagnetic waves to overcome the shortcomings of ultraviolet radiation. X-PACT (X-ray Psoralen Activated Cancer Therapy) exemplifies this by using uses low-dose X-rays (about 1 Gy) to activate inorganic phosphor nanoparticles. When activated, these nanoparticles emit UVA, enabling deeper tissue penetration and psoralen activation even within solid tumors (Oldham et al. 2016). Studies on the effectiveness of psoralen for treating solid tumors are currently underway in both canine and human subjects (Oldham et al. 2016).^1^ One current clinical trial (NCT04389281) has begun as a phase I study investigating X-PACT in solid tumors that do not exceed a depth of 5 cm (Footnote 1). To assure the photoactivation of psoralen, phosphor particles are paired with psoralen resulting in X-ray stimulated phosphorescence that then activates psoralen. The emission profile of phosphor overlaps the absorption and activation wavelengths of psoralen which has been previously confirmed (Oldham et al. 2016). Success of this approach would overcome the limited tissue penetration of ultraviolet radiation and potentially expand the clinical utility of psoralen beyond its established superficial use.

As the investigation into the treatment of deep-seated solid tumors continues, alternative delivery approaches for psoralen should be explored. Psoralen could be injected into malignant lymph nodes to study the strength of the antitumor immunoactivating effect. The injection could be performed using a fine needle aspiration needle under image guidance and may include unique approaches for more difficult to reach nodes. In the case of lung cancer, for example, malignant mediastinal and hilar nodes can often be easily and safely accessed via endobronchial ultrasound (EBUS) bronchoscopy. Injection of psoralen using a small needle into these malignant nodes can be performed under real-time ultrasound guidance and later activated by new photoactivating technologies. This concept holds promise for further exploration in the treatment of more difficult-to-treat malignancies. Of note, manual injection into the direct site is dependent on individual performance, and, thereby, subjected to intrinsic variability. More precise technology, such as nanotechnology (Keikha et al. 2021), could be considered in future to deliver the medication with more precision and accuracy.

### Autoimmune disease

Photoactivated psoralen has been utilized in the treatment of various autoimmune diseases, including the first published report on psoriasis. Psoriasis is a non-contagious, relapsing and chronic dermatological inflammatory skin disease. Current-treatment options for psoriasis include topical therapy, systemic therapy, biologics, phototherapy and PUVA (Sreya et al. 2023). Such ultraviolet-based therapies, with phototherapy and PUVA, impacts Langerhans cells, cytokines, and adhesion molecules, while inhibiting the proliferation of keratinocytes and angiogenesis, along with T cell apoptosis (Menter et al. 2010). Clinical trials conducted over the past 30 years have demonstrated the efficacy of PUVA therapy in psoriasis clearance, particularly when combined with other psoriasis-related medications such as acitretin and etretinate (Almutawa et al. 2013). Given the immunologic nature and superficiality of psoriasis, it is an ideal candidate for psoralen injection and activation by ultraviolet light, as deeper light penetration is not necessary.

Potential future investigations are currently underway to explore the role of psoralen pharmacotherapy in other autoimmune diseases. These investigations aim to impact the immunoproteasome, a multicatalytic protease expressed in cells of hematopoietic origin, as elevated expression has been associated with both cancer and autoimmune diseases (Schiffrer et al. 2019). Immunoproteasome compounds have psoralen-based rings that could be targeted to inhibit immunoproteasomes and assist with disease management (Schiffrer et al. 2019). In addition, there is potential to activate psoralen in deep-seated autoimmune phenomena such as granulomas, by utilizing X-rays instead of ultraviolet light. Furthermore, psoralen has been investigated for its potential to mitigate some of the side effects associated with TNF-alpha inhibitors, which are commonly used in the treatment of various autoimmune conditions. One specific side effect that has been targeted is muscle atrophy (Lin et al. 2020). However, this effect was observed in monolayer culture and therefore does not account for the barrier of light activation given tissue depth. More in vitro, animal, and ultimately human trials, are necessary to fully assess these potential benefits.

### Vascular malformations

Arteriovenous malformations and other vascular anomalies, especially those created by certain genetic predispositions, are the result of complex aberrant, angiogenic processes. Arteriovenous malformations are composed of dilated, thin-walled vascular networks, consisting of elastic fibers and smooth muscle cells, with the intima often thickened and partially covered by remnants of thrombi (Danyalian and Hernandez 2023). Arteriovenous malformations can form anywhere, from visceral organs (liver to lungs to brain), and can result in various complications depending on the organ they are impacting. Arteriovenous malformation management often consists of invasive treatments, from coiling to embolizing to surgical removal (Shovlin 2014; Faughnan et al. 2020). At times, these vascular anomalies cannot be intervened on with direct invasive procedures, warranting treatments that impact the angiogenic process of the vascular malformations. Systemic treatments include, for example, sirolimus, pazopanib, and bevacizumab (Galiatsatos et al. 2022; Adams et al. 2016; Faughnan et al. 2019). However, these systemic treatments carry side-effects and intolerabilities, along with an often life-long commitment to these pharmacotherapies.

UV-photoactivated psoralen has shown to have antiangiogenic properties. Specifically, clinical trials using PUVA have demonstrated the ability of photoactivated psoralen to downregulate the expression of vascular endothelial growth factor (VEGF), a protein known to stimulate the formation of blood vessels through tyrosine kinase pathways, and result in an aberrant formation of these vascular malformations in various diseases (Bethea et al. 1999; Longuet-Perret et al. 1998). For instance, VEGF is the key protein in vascular malformations in hereditary hemorrhagic telangiectasia (HHT), where established treatments such as bevacizumab directly target VEGF (Dupuis-Girod et al. 2012; Thompson et al. 2014). In vitro studies have shown PUVA to inhibit angiogenesis and induce apoptosis in endothelial cells as well (Deng et al. 2004). Therefore, a potential future application of photoactivated psoralen could be in the management of vascular malformations, with the use of ultraviolet light for superficial vascular anomalies, and X-rays for vascular anomalies in deeper, visceral organs.

### Advantage of psoralen UVA therapy

While currently there are therapies that established for treating the aforementioned diseases, psoralen’s main advantage may be in its side-effect profile when compared to other current treatments of chemotherapies, immunomodulators, surgeries, and/or radiation. Acute side-effects seem to be minimal when compared to these current cancer therapies. As for long-term concerns, while psoralen has been linked with the genesis of cancer, it appears to be associated via a dose-dependent response (Ren et al. 2020). However, what dose specifically yields such a higher risk of malignancy is unknown, and further monitoring of the treatment is warranted to establish a dose-responsive curve for this issue. In addition to the relative side-effect profile, an additional advantage of psoralen is the candidacy for patients who may be poor candidates for current standard of care therapies. Therefore, offering patients more of an option for treatments.

Of note, there are potential other photosensitizers, such as psoralen derivatives, that may be more advantageous given methoxsalen’s limitations in its ability as a producer of singlet oxygen. For example, synthetic derrivatives of psoralen such as sulfur and selenium analogs have shown different photosensitizing properties compared to methoxsalen, in a manner where there are more efficient (Seret et al. 1992). Specifically, these new synthetic derivatives have a significant singlet oxygen quantum yields, and likely, which may prove more advantageous as a therapy (Seret et al. 1992; Egorov et al. 1999). Future research should explore these psoralen derivatives in the clinical setting.

### Ex vivo applications

Psoralen can also be used in various ex vivo applications. One example is that of the Helinx technology which utilizes the synthetic psoralen compound, amotosalen hydrochloride, and UVA light to inactivate infectious pathogens and leukocytes in platelets and plasma for blood banks (Wollowitz 2001). The process involves amotosalen intercalating into nucleic acids, forming a monoadduct with UVA light, and finally creating an interstrand crosslink to inhibit replication. This technology is particularly effective against larger genomes, like those in leukocytes, and thus has application for pathogen inactivation for platelets and plasma for blood-bank use (Wollowitz 2001). Using a similar approach, synthetic psoralens can also be used to limit the proliferation of naïve and primed donor T cells thus decreasing the potential for graft versus host disease after donor leukocyte infusions (Truitt et al. 1999).

## Conclusion

Psoralen demonstrates significant clinical potential, primarily due to its cytotoxicity when activated by specific frequencies of electromagnetic radiation, such as UVA and low-dose X-rays. The photoreactivity of psoralen results in mono- and di-adducts with DNA, leading to marked cell apoptosis. This apoptotic effect is specifically targeted towards pathologic cells with high rates of cell division, such as those seen in malignancy and certain autoimmune processes. Furthermore, photoactivated psoralen may block tyrosine kinase signaling and influence the immunogenic properties of cells, which may have significant clinical applications for cancer, autoimmune disorders, and vascular malformations. While photoactivated psoralen treatment using ultraviolet light is well established for superficial conditions, exploring its clinical utility with other technologies, such as low-dose X-rays and phosphor nanoparticles, holds promise for potential applications targeting more deeply seated pathologies, such as solid tumors. The potential of photoactivated psoralen to increase the therapeutic ratio of treatment across a variety of diseases is likely to garner increased attention over the next decade with a resulting increase in its clinical utilization.

## Data Availability

No datasets were generated or analysed during the current study.

## Footnotes

1 Clinicaltrials.gov. X-ray psoralen activated cancer therapy in head and neck, breast, sarcoma and melanoma—NCT04389281. https://classic.clinicaltrials.gov/ct2/show/NCT04389281. Accessed 4 July 2023.
